# Comparison of anterior and posterior trabecular bone microstructure of human mandible using cone-beam CT and micro CT

**DOI:** 10.1186/s12903-021-01595-z

**Published:** 2021-05-08

**Authors:** Norliza Ibrahim, Azin Parsa, Bassam Hassan, Paul van der Stelt, Rabiah A. Rahmat, Siti M. Ismail, Irene H. A. Aartman

**Affiliations:** 1 Department of Oral and Maxillofacial Clinical Sciences, Faculty of Dentistry, University of Malaya, 50603 Kuala Lumpur, Malaysia; 2 Department of Diagnostic Sciences, School of Dentistry, The University of North Carolina, Chapel Hill, USA; 3 Department of Oral and Maxillofacial Radiology, Academic Center for Dentistry Amsterdam (ACTA), Louwesweg 1, 1066 EA Amsterdam, The Netherlands

## Abstract

**Background:**

The aim of this study was to compare the trabecular bone microstructures of anterior and posterior edentulous regions of human mandible using cone-beam computed tomography (CBCT) and micro computed tomography (µCT).

**Methods:**

Twenty volumes of interests consisting of six anterior and fourteen posterior edentulous regions were obtained from human mandibular cadavers. A CBCT system with a resolution of 80 µm (3D Accuitomo 170, J. Morita, Kyoto, Japan) and a µCT system with a resolution of 35 µm (SkyScan 1173, Kontich, Belgium) were used to scan the mandibles. Three structural parameters namely, trabecular number (Tb.N), trabecular thickness (Tb.Th), and trabecular separation (Tb.Sp) were analysed using CTAn software (v 1.11, SkyScan, Kontich, Belgium). For each system, the measurements obtained from anterior and posterior regions were tested using independent sample *t*-test. Subsequently, all measurements between systems were tested using paired *t-*test.

**Results:**

In CBCT, all parameters of the anterior and posterior mandible showed no significant differences (*p* > 0.05). However, µCT showed a significant different of Tb.Th (*p* = 0.023) between anterior and posterior region. Regardless of regions, the measurements obtained using both imaging systems were significantly different (*p* ≤ 0.021) for Tb.Th and Tb.N.

**Conclusions:**

The current study demonstrated that only the variation of Tb.Th between anterior and posterior edentulous region of mandible can be detected using µCT. In addition, CBCT is less feasible than µCT in assessing trabecular bone microstructures at both regions.

## Introduction

Cone-beam computed tomography (CBCT) is used in clinical dentistry to evaluate both the bone geometry [1] and bone density [2] mainly due to its advantages in comparison with other 3D imaging modalities [3]. However, the bone density assessment in CBCT images are not consistent as in medical CT [4]. Along with the advancement of the CBCT scanning resolution, studies on trabecular bone microstructure using CBCT is becoming more available [5–9]. Furthermore, the accuracy of CBCT [5, 9] and the influence of its scanning parameters for trabecular bone microstructure assessment have been explored [7–9].

Micro computed tomography (µCT) has largely been used to analyze structural measurements of bones. Previous µCT studies showed variation in the bone microstructure measurements, depending on the site and the density of the samples [10–12]. Due to limited clinical applications, the assessment of trabecular microstructures for oral maxillofacial region cannot be conducted in vivo using µCT [13]. In this context the use of high resolution CBCT appears promising [3]. Therefore, it is worth to investigate the potential of CBCT in detecting the variations of trabecular microstructures at different bony maxillofacial regions. Human mandibular bone demonstrates a denser bone trabeculation at the anterior region in comparison to the posterior region [14]. Most bone quality studies related to dental implant are mainly limited to bone density [14] and quantity assessment [15].

Bone quality can be better assessed by measuring both bone density and trabecular microstructure parameters [13, 16–18]. Trabecular microstructure has been reported as one of the determinants to predict primary implant stability [19, 20], bone healing, osseointegration [21] and bone strength [22]. Thus, the aims of this study were of two-fold: (1) to compare trabecular bone microstructure parameters between anterior and posterior edentulous human mandible using CBCT and µCT; (2) to evaluate the difference of CBCT and µCT in measuring trabecular bone microstructure at anterior and posterior regions of edentulous mandible.

## Materials and methods

Twenty-five human mandibular cadavers were obtained from the Department of Functional Anatomy, Academic Center for Dentistry Amsterdam, and approved for research purposes. The inclusion criteria for human mandibular cadavers are edentulous posterior and/or anterior, no mandibular developmental anomaly, and no associated pathological conditions. The mandibles were scanned using a CBCT system with a resolution of 80 μm (3D Accuitomo 170, J. Morita, Kyoto, Japan). The scan protocol for CBCT consisted of a 4⋅4 cm FOV using a high-resolution scan mode and a full rotation (360°). CBCT images were acquired at 90 kVp, 5.0 mA and 17.5 s exposure time. After the scanning, twenty edentulous regions of the mandibles (6 anterior and 14 posterior) were selected to be included in this study. Subsequently, the mandibles were re-scanned using a µCT system with a resolution of 35 μm (SkyScan 1173, Kontich, Belgium). During the µCT scanning, the mandibles were secured in a cylindrical styrofoam and mounted to the holder. µCT images were acquired at 130 kVp and 61 mA. The images from both systems were exported as Digital Imaging and Communications in Medicine (DICOM 3) files and imported into an image analysis software (Amira v4.1, Visage Imaging Inc., Carlsbad, CA).

**Fig. 1:**
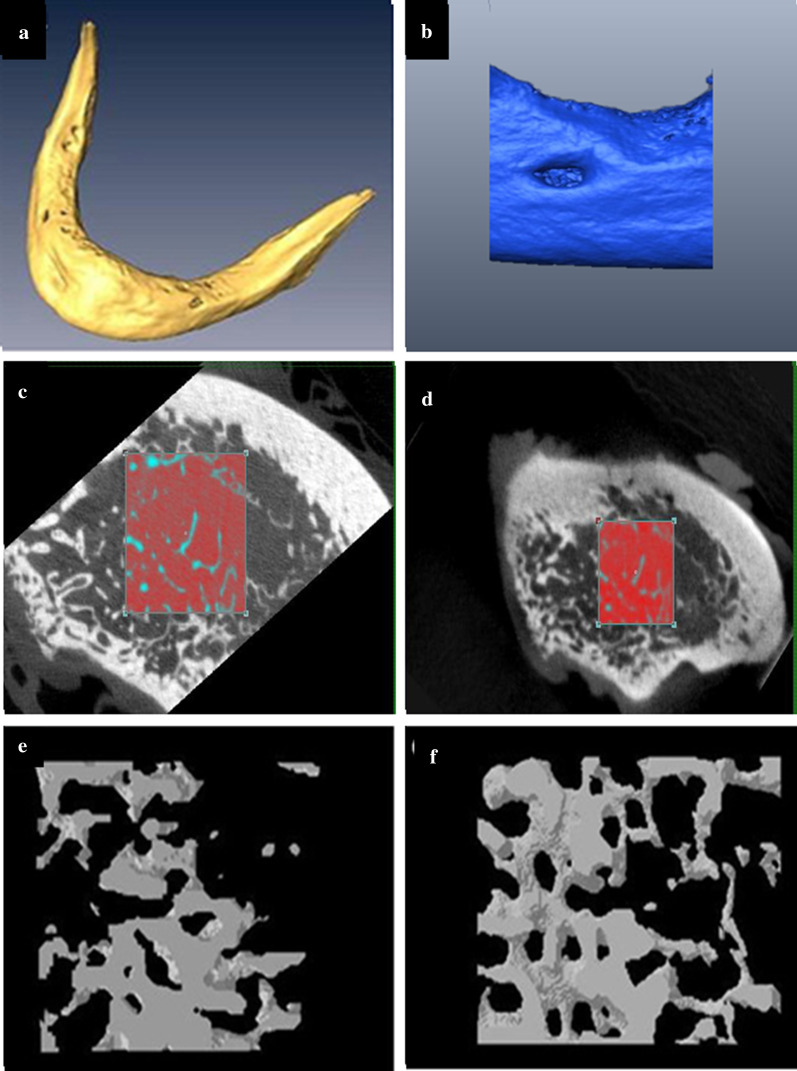
Comparison of trabecular bone microstructures of human edentulous mandible using CBCT and µCT. **a** 3D CBCT image of an edentulous mandible. **b** 3D µCT image of a segmented edentulous mandible. Step of matching and comparing the anatomical landmark from the VOI of CBCT (**c**) and µCT (**d**). Trabecular bone microstructure of CBCT (**e**) and µCT (**f**) was analysed using CTAn v. 1.11 software (SkyScan, Kontich, Belgium)

Volume of interests (VOIs) were identified based the following criteria: the edentulous region must not be less than 5 mm in length and not associated with any metallic artifact. In total, twenty VOIs of the edentulous regions were segmented and compared. Then, a surface-based image registration process was performed to ensure that the CBCT’s and micro-CT’s VOIs were taken from the same region (Fig. 1a, b). The measurement of trabecular microstructure was obtained by importing the selected VOIs into an image structural analysis software CTAn (v 1.11, SkyScan, Kontich, Belgium) as 16-bitmap (BMP) images (65,536 Gy values). Next, to further ensure the measurements were from the same region, an additional step of matching and comparing the anatomical landmark from the VOI of CBCT (Fig. 1c) and micro-CT (Fig. 1d) was performed. An automated thresholding method was used to binarize the datasets to obtain the measurement of trabecular number (Tb.N), trabecular thickness (Tb.Th) and trabecular separation (Tb.Sp) (Fig. 1e, f). Throughout the analysis, the images were viewed using a 22-inch computer monitor (full high-definition 1920 × 1080 pixel; Dell, Texas, United States) in a quiet and dimmed light room. All measurements were performed twice with an interval of two weeks by one trained maxillofacial radiologist with more than ten years of experience evaluating CT images.

### Statistical analysis

Data were analyzed using SPSS® (v20.0, SPSS Inc., IBM Corp., Armonk, NY). Intraclass correlation coefficient (ICC) was used to assess the intraobserver’s reliability. Independent sample *t*-test was performed to assess the measurement difference of each trabecular parameters (Tb.N, Tb.Th, Tb.Sp) between anterior and posterior VOIs of CBCT and µCT respectively. Paired *t*-tests were used to assess the difference between CBCT and µCT measurements. The level of significance was set at *p* = 0.05.

## Results

The ICC tests revealed excellent intraobserver reliability for all parameters and both systems (CBCT ≥ 0.96, µCT ≥  0.99).

### Comparison of trabecular microstructure parameters between anterior and posterior region

Table 1 shows the descriptive statistics and test results of the trabecular bone microstructure measurements between anterior and posterior mandibular regions using CBCT and µCT. All measurements obtained from CBCT showed no significant differences between edentulous anterior and posterior regions parameters were not significant when analyzed using CBCT (*p* ≥ 0.09). Similarly, µCT showed no significant difference (Tb.N, *p* ≥ 0.580 and Tb.Sp, *p ≥* 0.381) for all parameters, except for Tb.Th (*p* = 0.023).

**Table 1:** Inter-regional comparison of trabecular microstructure parameters using CBCT and µCT. The measurements of trabecular number (Tb.N), trabecular thickness (Tb.Th) and trabecular separation (Tb.Sp) were compared between anterior (n = 6) and posterior (n = 14) edentulous regions using the independent sample t-test.)

Systems	Parameters	Region	Mean	SD	SEM	t	df	*p*
CBCT	Tb.N (μm^−1^)	Anterior	5.91	1.67	0.68	0.21	18	0.84
		Posterior	5.60	3.33	0.89			
	Tb.Th (μm)	Anterior	7.19	1.78	0.73	1.78	18	0.09
		Posterior	5.63	1.81	0.48			
	Tb.Sp (μm)	Anterior	9.47	2.68	1.10	− 0.20	18	0.85
		Posterior	9.81	3.71	0.99			
µCT	Tb.N (μm^−1^)	Anterior	7.96	2.50	1.02	0.56	18	0.58
		Posterior	6.95	4.03	1.08			
	Tb.Th (μm)	Anterior	4.84	0.78	0.32	2.48	18	0.02*
		Posterior	3.64	1.07	0.29			
	Tb.Sp (μm)	Anterior	7.39	1.51	0.62	− 0.90	18	0.38
		Posterior	9.07	4.42	1.18			

*Significant difference was accepted at *p* < 0.05

### Comparison of trabecular microstructure parameters between CBCT and µCT

All parameters of trabecular bone microstructures measurements between CBCT and µCT were significantly different (*p* ≤ 0.021), except for Tb.Sp (*p* = 0.180) as shown in Table 2. At both regions, the Tb.N average measurement was lower in CBCT (anterior = 5.61 μm^−1^, posterior = 5.60 μm^−1^) compared to µCT (anterior = 7.96 μm^−1^, posterior = 6.95 μm^−1^. In contrast, Tb.Sp and Tb.Th were higher in CBCT (Tb.Sp anterior = 9.47 μm, Tb.Sp posterior = 9.81 μm; Tb.Th anterior = 7.19 μm, Tb.Th posterior = 5.63 μm) than µCT (Tb.Sp anterior = 7.39 μm, Tb.Sp posterior = 9.07 μm; Tb.Th anterior = 4.84 μm, Tb.Th posterior = 3.64 μm).

**Table 2:** Intra-regional comparison of trabecular microstructure parameters using CBCT and µCT. The measurements of trabecular number (Tb.N), trabecular thickness (Tb.Th) and trabecular separation (Tb.Sp) of either anterior (n = 6) or posterior (n = 14) regions were compared using the independent sample *t*-test

Parameters	Regions	CBCT			µCT			*t*-test		
		Mean	SD	SEM	Mean	SD	SEM	t	df	*p*
Tb.N (μM^−1^)	Anterior	5.61	1.67	0.68	7.96	2.50	1.02	3.46	5	0.018*
	Posterior	5.60	3.33	0.89	6.95	4.03	1.08	4.21	13	0.001*
Tb.Th (μM)	Anterior	7.19	1.78	0.73	4.84	0.78	0.32	− 4.73	5	0.005*
	Posterior	5.63	1.81	0.48	3.64	1.07	0.29	− 4.90	13	0.001*
Tb.Sp (μM)	Anterior	9.47	2.68	1.10	7.39	1.51	0.62	− 3.33	5	0.021*
	Posterior	9.81	3.71	0.99	9.07	4.42	1.18	− 1.42	13	0.180

*Tb.N* trabecular number, *Tb.Th* trabecular thickness, *Tb.Sp* trabecular spacing, *Ant* anterior, *Post* posterior, *SD* standard deviation, *SEM* standard error of mean

*The difference is significant at *p* < 0.05. Significant difference was accepted at *p* < 0.05

## Discussion

Trabecular microstructure is one of the important determinants for bone quality. The latest CBCT generation offers a high scanning resolution which is adequate for trabecular microstructural evaluation [3, 4, 6, 23]. Prior to its application for clinical evaluation, the accuracy of CBCT measurements have been compared to a reference modality i.e. µCT [3, 5, 6, 9]. However, most CBCT based studies are constrained to the technical influence of various scanning parameters [6–8]. Assessment of the regional bone quality variations is important in predicting the success of implant treatment at different sites of human mandible [14, 15]. Kim et al. [24] demonstrated the microstructural differences between various regions of maxilla and mandible. However, the study was limited to µCT and dentate regions. To our knowledge, this is the first study assessing microstructural bone parameters between anterior and posterior edentulous regions of mandible using CBCT.

Trabecular bone varies according to the mandibular regions [24–26]. This is due to the disparity of complex trabecular configurations [17, 27]. Unlike µCT, this current study demonstrated that Tb.Th measurement was not significantly different in CBCT. However, it was the only the potential parameter to distinguish both regions (Table 1). In contrast, other µCT and histomicromorphic studies [17, 27] have found significant differences in more than one parameter. This might be due the differences in bone density [10, 16, 17], type of specimens [17], scanning protocols [7] and the system’s technology [28] used in this study.

Microstructural evaluation is highly dependent on the image resolution [29–31]. The current study demonstrated that structural measurements obtained from both CBCT (80 μm) and µCT (35 μm) were different, except for Tb.Sp at posterior region (Table 2). This result was concordance to a µCT study reported by Fanuscu and Chang [17] that used different resolution (12–110 μm). Although a small voxel size (< 100 μm) is recommended for microstructural evaluation [10, 17, 31], the resolution for an accurate analysis is still dependent on the bone origin [11, 32] and regional density [17]. It was described that a low-density bone may exhibit a wide variation of structural measurements in regards to the thresholding technique imposed [16]. Thus, the trabecular structural parameters at different density regions might be over- or under-estimated resulting in unfavorable differences in this study.

Since this study was the first of its kind, the optimum resolution for CBCT microstructural assessment was not available in the literature. Therefore, a specific resolution should be set when comparing different types of bone density in future studies. This study has only assessed the difference of trabecular bone microstructure at two different regions of edentulous mandibles. Hence, further studies should be conducted to assess maxillary regions with different bone density, scanning protocols and the system’s technology.

## Conclusions

The current study has suggested that micro CT can depict the differences of Tb.Th between the anterior and posterior edentulous regions of mandible. The use of CBCT is less feasible due to inadequate resolution in depicting structural differences at different regions.

## Data Availability

The datasets generated during and analysed during the current study are not publicly available because it contains personal information but are available from the first author on reasonable request.
